# Risk Factors for Hospital Readmission Post-Transcatheter Aortic Valve Implantation in the Contemporary Era: A Systematic Review

**DOI:** 10.1016/j.cjco.2022.05.007

**Published:** 2022-06-06

**Authors:** Raumil V. Patel, Mithunan Ravindran, Ragavie Manoragavan, Abi Sriharan, Harindra C. Wijeysundera

**Affiliations:** a Temerty Faculty of Medicine, University of Toronto, Toronto, Ontario, Canada; b Institute for Health Policy, Management, and Evaluation, University of Toronto, Toronto, Ontario, Canada; c Division of Cardiology, Department of Medicine, Schulich Heart Program, Sunnybrook Health Sciences Centre, Toronto, Ontario, Canada; d Sunnybrook Research Institute, University of Toronto, Toronto, Ontario, Canada; e Institute for Clinical Evaluative Sciences, Toronto, Ontario, Canada

## Abstract

**Background:**

Despite transcatheter aortic valve implantation (TAVI) becoming a widely accepted therapeutic option for the management of aortic stenosis, post-procedure readmission rates remain high. Rehospitalization is associated with negative patient outcomes, as well as increased healthcare costs, and has therefore been identified as an important target for quality improvement. Strategies to reduce the post-TAVI readmission rate are needed but require the identification of patients at high risk for rehospitalization. Our systematic review aims to identify predictors of post-procedure readmission in patients eligible for TAVI.

**Methods:**

We conducted a comprehensive search of the MEDLINE, Embase, and Cochrane Central Register of Controlled Trials (CENTRAL) databases for the time period from 2015 to the present for articles evaluating risk factors for rehospitalization post-TAVI with a follow-up period of at least 30 days in adults age ≥ 70 years with aortic stenosis. The quality of included studies was evaluated using the Newcastle-Ottawa Scale. We present the results as a qualitative narrative review.

**Results:**

We identified 49 studies involving 828,528 patients. Post-TAVI readmission is frequent, and rates vary (14.9% to 54.3% at 1 year). The most-frequent predictors identified for both 30-day and 1-year post-TAVI readmission are atrial fibrillation, lung disease, renal disease, diabetes mellitus, in-hospital life-threatening bleeding, and non-femoral access.

**Conclusions:**

This systematic review identifies the most-common predictors for 30-day and 1-year readmission post-TAVI, including comorbidities and potentially modifiable procedural approaches and complications. These predictors can be used to identify patients at high-risk for readmission who are most likely to benefit from increased support and follow-up post-TAVI.

## Résumé

**Contexte:**

Bien que l’implantation valvulaire aortique par cathéter (IVAC) soit maintenant une option thérapeutique largement acceptée pour la prise en charge de la sténose aortique, les taux de réadmission des patients après cette intervention demeurent élevés. La réhospitalisation est associée à des résultats de santé défavorables pour les patients ainsi qu’à des coûts de soins de santé plus élevés, ce qui en fait une cible importante pour l’amélioration de la qualité des soins. Des stratégies de réduction des taux de réadmission après l’IVAC sont nécessaires, mais elles exigent de repérer les patients qui présentent des risques de réhospitalisation plus élevés. Notre revue systématique vise à cerner les facteurs prédictifs de réhospitalisation après l’intervention chez les patients admissibles à une IVAC.

**Méthodologie:**

Nous avons effectué une recherche exhaustive dans les bases de données MEDLINE, Embase et Cochrane Central Register of Controlled Trials (CENTRAL) pour trouver les articles publiés entre 2015 et aujourd’hui qui rapportent les facteurs de risque de réhospitalisation suite à une IVAC chez les adultes âgés de 70 ans et plus atteints de sténose aortique, avec une période de suivi d’au moins 30 jours. La qualité des études retenues a été évaluée à l’aide de l’échelle Newscastle-Ottawa. Les résultats sont présentés sous forme d’une revue narrative qualitative.

**Résultats:**

Nous avons retenu 49 études, réalisées auprès de 828 528 patients. La réhospitalisation après l’IVAC était fréquente, et les taux étaient variables (de 14,9 % à 54,3 % après un an). Les facteurs prédictifs de réhospitalisation les plus fréquents, déterminés 30 jours et un an après l’IVAC, étaient la fibrillation auriculaire, la maladie pulmonaire, la maladie rénale, le diabète, l’hémorragie menaçant le pronostic vital lors du séjour à l’hôpital, et l’approche par une voie non fémorale.

**Conclusions:**

La présente revue systématique nous a permis de caractériser les facteurs prédictifs les plus fréquents de réhospitalisation, 30 jours et un an après une IVAC, dont certaines affections concomitantes et certains facteurs potentiellement modifiables liés aux approches d’intervention et aux complications. Ces facteurs pourraient permettre de cibler les patients à risque élevé de réhospitalisation, qui seraient les plus susceptibles de bénéficier d’un soutien et d’un suivi accrus après une IVAC.

The prevalence of aortic stenosis, the most common form of valvular heart disease, is expected to grow significantly as the population ages, representing a major challenge to healthcare systems around the world.^1^ In Canada, transcatheter aortic valve implantation (TAVI) has become the standard of care for inoperable or high-surgical-risk patients with symptomatic severe aortic stenosis,^1^ with more recent evidence supporting use of TAVI as a reasonable alternative in intermediate- and low-risk patients.^2^^,^^3^ Despite the dramatic improvements in the safety and efficacy of TAVI, post-procedure early and late readmissions remain a concern. Recent studies have reported that up to 22.4% and 54.3% of patients are readmitted at 30 days and 1 year after TAVI, respectively.^4^^,^^5^

Preventing avoidable rehospitalizations has emerged as an important quality-improvement initiative, with policies such as the Hospital Readmissions Reduction Program in the US offering financial incentives to minimize unnecessary hospitalizations.^6^

In TAVI, both cardiac and noncardiac causes have been identified as important contributors.^5^^,^^7^ Rehospitalization has been associated with an increase in all-cause mortality^8^ and healthcare costs.^9^ Reducing readmission rates requires a rigorous means of identifying patients at high risk for readmission. Accordingly, in this systematic review, we aim to summarize the current evidence on predictors of post-procedure readmission in patients with aortic stenosis eligible for TAVI, with the goal of identifying pre-procedural comorbidities, as well as potentially modifiable peri-procedural predictors. This identification in turn would facilitate interventions to reduce readmissions, which would need to be independently tested.

## Methods

### Protocol and registration

Our systematic review is reported in accordance with the **P**referred **R**eporting **I**tems for **S**ystematic Reviews and **M**eta-**A**nalyses (PRISMA) statement,^10^ and registered with the International Prospective Register of Systematic Reviews (PROSPERO: CRD42021244168).

### Eligibility criteria

We included randomized controlled trials (RCTs), observational cohort studies, and retrospective case-control studies of adults (age ≥70 years) with aortic stenosis eligible for TAVI. We excluded studies in which the primary intervention was surgical aortic valve replacement or valve-in-valve TAVI.

To be included, a study had to evaluate one or more risk factors for rehospitalization post-TAVI, with a follow-up period of at least 30 days. Our primary outcome of interest is risk factors for early (at < 30 days) and late (at ≥30 days) all-cause readmission post-TAVI, and secondary outcomes include cardiac vs noncardiac causes of both early and late hospital readmission rates, as well as all-cause mortality in readmitted patients.

We excluded case reports, conference abstracts, and review articles. We included only studies written in the English language, and only those published in 2015 or later, as this period saw the expansion of TAVI to intermediate- and low-risk patients.

### Information sources

The databases, platforms, and coverage were MEDLINE, Embase, and the Cochrane Central Register of Controlled Trials (CENTRAL), from 2015 to present.

### Search strategy

The search strategy was developed by a medical librarian in consultation with a study investigator (R.P.). An exploratory literature review was conducted to find relevant articles, to mine key words. The search strategy was formed based on the following key words: “transcatheter aortic valve implantation,” “transcatheter aortic valve replacement,” “readmission,” and “rehospitalization.” The following medical subject heading (MeSH) terms were exploded: “transcatheter aortic valve replacement,” “heart valve prosthesis implantation,” “patient readmission,” and “hospital readmission.” The full search strategies are available in Supplemental Table S1.

### Selection process

After de-duplication of search results, 2 independent investigators (R.P. and R.M.) used predefined inclusion and exclusion criteria to review titles and abstracts. Afterward, the full texts of eligible studies were reviewed for inclusion by 2 independent investigators (R.P. and M.R.). Throughout the screening process, all disagreements were resolved via consensus or via a third investigator (H.W.) when consensus could not be reached. De-duplication, screening, and data abstraction were performed using the Web-based Covidence software (Veritas Health Innovation, Melbourne, Australia).^11^

### Data collection process

We prepared a data extraction form on Covidence, and 2 independent investigators (R.P. and M.R.) carried out data extraction from eligible studies. The 2 investigators met to identify and resolve discrepancies through discussion. Please see Supplemental Table S2 for data elements.

### Variables

In studies for which both univariate and multivariable analyses were performed to determine predictors for readmission, we included only predictors that were statistically significant in the multivariable analysis. We categorized predictors for early and late hospital readmission into 7 categories: (i) demographic factor; (ii) clinical characteristic; (iii) cardiac comorbidity or previous intervention; (iv) medical comorbidity; (v) laboratory marker; (vi) procedural characteristic; and (vii) procedural complication. Cardiac causes were those pathologies that are intrinsic to the heart, such as arrhythmia or heart failure, whereas noncardiac causes were all other medical comorbidities, such as lung or renal pathology. Procedural characteristics and complications were considered to be potentially modifiable. The Elixhauser Comorbidity Index quantifies a patient’s comorbidity based on 30 International Classification of Diseases (ICD), ninth revision, Clinical Modification and ICD, tenth revision diagnosis codes, which are weighted based on the association of each comorbidity with death, to produce a summary index.^12^

### Study quality assessment

Two authors (R.P. and M.R) independently evaluated study quality using the Newcastle-Ottawa Scale, and discrepancies were resolved by discussion. The quality assessment is presented in Supplemental Table S3.

### Statistical analysis

We extracted the absolute number of events when it was available, or calculated it based on the statistical measures reported. Given the substantial heterogeneity among the included studies in the statistical measures used to report outcome data, a meta-analysis was not performed.

## Results

### Study selection (flow of studies)

**Figure 1:**
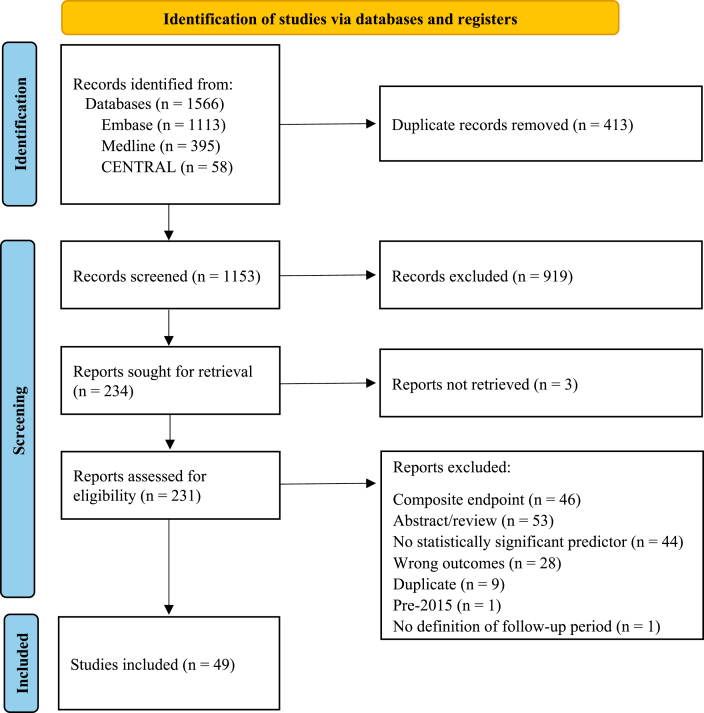
**P**referred **R**eporting **I**tems for **S**ystematic Reviews and **M**eta-**A**nalyses (PRISMA) flow diagram showing study selection. CENTRAL, Cochrane Central Register of Controlled Trials.

Figure 1 illustrates the **P**referred **R**eporting **I**tems for **S**ystematic Reviews and **M**eta-**A**nalyses (PRISMA) flow diagram for study selection. Database searching retrieved 1566 records, of which 413 were duplicates. We excluded 919 records after title and abstract screening, and assessed the full texts of 231 records. Ultimately, we included 49 studies in the review.

### Study characteristics

Study characteristics are summarized in Table 1. The design of all included studies was either cohort (45) or RCT (4), and all were published between 2015 and 2022. The study period for included studies ranged from 1 to 10 years. Seventeen of the included studies were reports on early hospitalizations, 21 were reports on late hospitalizations, and 11 were reports on both. The definitions of early and late follow-up periods were similar across studies. Across all included studies, 828,528 patients underwent TAVI, and sample sizes ranged from 63 to 171,361 patients. The percentage of female patients ranged from 27% to 69.8% across studies. Study patients were older adults (age 74-84 years). Only 18 studies reported baseline surgical risk. Most patients were at intermediate or significant risk for in-hospital mortality and morbidity. The Society of Thoracic Surgeons (STS) score, the **Euro**pean **S**ystem for **C**ardiac **O**perative **R**isk **E**valuation (EuroSCORE), and the EuroSCORE II ranged from 3.2% to 10%, 13.3% to 23%, and 0.07% to 5.9%, respectively. Of the 15 studies reporting access site, the transfemoral approach was most frequently used, with the percentage of patients having a transfemoral TAVI ranging from 45% to 97.4%. The type of valve used was reported by 13 studies. Balloon-expandable valves were most common (6% to 100%), followed by self-expandable valves (0% to 90%).

**Table 1:** Characteristics of included studies

Study	Study design	Study period, y	Follow-up period	Population size	Average age, y	Female patients	STS score	EuroScore	EuroScore II	Transfemoral access	Balloon-expandable valve	Self-expandable valve	Other valve
Arai et al. 2018^13^	C	3	B	1215	—	—	—	—	—	—	—	—	—
Auffret et al. 2020^16^	C	8	B	750	—	—	—	—	—	—	—	—	—
Czarnecki et al. 2019^28^	C	6	L	937	83 (78–87)	44.3	—	—	0.07 ± 0.06	73.4	53.6	44.2	2.2∗
Czarnecki et al. 2020^15^	C	4	B	2547	83 (78–87)	45.7	—	—	—	86.4	45.4	32.6	7.9
Dodson et al. 2017^23^	C	4	E	18,568	84 (79–88)	48.6	6.8 (4.5–10.2)	—	—	67.2	—	—	—
Durand et al. 2017^17^	C	4	L	546	83.9 ± 7.3	53.3	—	15.6 ± 10.9	—	87.8	97.8	2.2	0
Elbaz-Greener et al. 2019^29^	C	4	B	2129	83 (78–87)	45.8	—	—	—	81.6	—	—	—
Forcillo et al. 2017^18^	C	8	B	714	83 (77–87)	46.6	10 (7.2–13.9)	—	—	58.8	100	0	0
Franzone et al. 2017^8^	C	7	L	868	82.4±5.8	53.7	6.6 ± 4.3	21.4	—	—	—	—	—
Guedeney et al. 2019^19^	C	6	L	1139	82.4 ± 7.7	47.8	4.3 ± 3.1	—	5.2 ± 4.5	82.4	60.7	39.3	0
Kolte et al. 2017^14^	C	1	E	12,221	81.5 ± 8.4	49.1	—	—	—	—	—	—	—
Nombela-Franco et al. 2015^5^	C	-	B	720	82 (77–86)	58.2	—	16.6 (10.1–25)	—	66.5	84.6	15.4	0
Panaich et al. 2016^4^	C	1	E	5702	—	49.6	—	—	—	—	—	—	—
Sanchez et al. 2020^30^	C	2	E	10,345	81.2 ± 7.9	62.5	7.5 ± 4.9	—	—	—	—	—	—
Tripathi et al. 2020^31^	C	1	L	73,784	—	45.5	—	—	—	97.4	—	—	—
Yerasi et al. 2021^32^	C	4	E	3104	80.3 ± 8.4	39	—	—	—	—	—	—	—
Doshi et al. 2019^24^	C	3	E	54,117	—	46.9	—	—	—	83.16	—	—	—
Pajjuru et al. 2022^33^	C	5	L	171,361	—	53.5	—	—	—	—	—	—	—
Johansson et al. 2016^34^	C	6	L	166	—	49	—	23 ± 15	—	45	0	90	10
Malik et al. 2020^35^	C	1	L	20,504	80.6 ± 8.3	45.9	—	—	—	—	—	—	—
Saji et al. 2018^36^	C	3	B	155	85 (82–88)	65	6 (4.7–8.2)	—	—	74	91	8.3	0.7
Deharo et al. 2020^37^	C	10	L	31,113	—	—	—	—	—	—	65.6	34.4	0
Ko et al. 2018^38^	C	3	L	63	81.7 ± 7.6	47	—	—	—	—	6	81	13
Aljabbary et al. 2018^39^	C	5	L	1257	82.3 ± 7.2	47.1	—	—	—	—	—	—	—
Chamandi et al. 2018^40^	C	8	L	1629	—	—	—	—	—	—	—	—	—
Nazif et al. 2015^41^	RCT	10	L	1973	—	—	—	—	—	—	—	—	—
Jorgensen et al. 2019^42^	C	10	L	816	81 (75–85)	—	3.2 (2.2–4.9)	—	—	—	—	—	—
Nazif et al. 2019^43^	RCT	5	L	1179	—	—	—	—	—	—	—	—	—
Doshi et al. 2020^44^	C	3	E	10,847	82.4 ± 7.2	46.4	—	—	—	—	—	—	—
Mentias et al. 2019^45^	C	2	L	72,660	81.9 ± 8.1	47	—	—	—	—	—	—	—
Zweiker et al. 2017^46^	C	7	L	398	82 (78–85)	63	6.3 (3.8–9.6)	13.3 (7.8–23.8)	5.9 (3.2–10.8)	—	—	—	—
Shahim et al. 2021^47^	RCT	4	L	948	—	—	—	—	—	—	—	—	—
Hioki et al. 2017^48^	C	3	L	1124	85 (82–88)	69.8	6.7 (4.7–9.4)	—	—	78.8	—	—	—
Caughron et al. 2021^49^	C	6	B	309	78.2 ± 10.3	44.3	5 ± 4.4				75.7	24.3	0
Ando et al. 2020^50^	C	4	E	5731	74 ± 10.1	38.1	—	—	—	—	—	—	—
Feldman et al. 2021^51^	C	3	L	341	81.4 ± 8	51.9	6.7 ± 4.8	—	—	86.8	—	—	—
Gracia et al. 2020^52^	C	3	E	298	—	—	—	—	—	—	—	—	—
Testa et al. 2016^53^	C	4	B	990	—	—	—	—	—	—	—	—	—
Thourani et al. 2016^54^	RCT	10	B	2531	—	—	—	—	—	—	—	—	—
Tirado-Conte et al. 2016^55^	C	1	L	303	84 (79–87)	63	—	—	3.62 (2.6–6)	—	67	33	0
O'Leary et al. 2020^56^	C	8	L	3391	82 ± 7.5	41.9	—	—	—	—	—	—	—
Lemor et al. 2019^57^	C	3	E	36,269	81.3 ± 8.5	47.9	—	—	—	—	—	—	—
Inohara et al. 2018^58^	C	2	L	21,312	—	—	—	—	—	—	—	—	—
Hermann et al. 2018^59^	C	3	L	62,125	82 (76–87)	46.3	6 (3.9–9.3)	—	—	—	—	—	—
Emami et al. 2020^60^	C	5	E	105,603	—	—	—	—	—	—	—	—	—
Arora et al. 2020^61^^,^^62^	C	5	E	47,255	—	—	—	—	—	—	—	—	—
Freitas-Ferraz et al. 2020^63^	C	6	L	308	80.5 ± 7.2	27	7.7 (5.3–11.9)	—	—	71.4	80.8	—	—
McCarthy et al. 2018^64^	C	4	B	34,576	—	—	—	—	—	—	—	—	—
Miura et al. 2020^65^	C	3	L	1587	—	—	—	—	—	—	—	—	—

Values are %, unless otherwise indicated.

B, both; C, cohort; E, early; EuroSCORE, **Euro**pean **S**ystem for **C**ardiac **O**perative **R**isk **E**valuation; L, late; RCT, randomized controlled trial; STS, Society of Thoracic Surgeons.

∗ Calculated from study data.

### Readmission rates

Only 17 of the 49 included studies reported readmission rate data (Table 2). Readmission rates were divided into 2 categories—early and late—and 3 subcategories—all-cause, cardiac cause, and noncardiac cause. Post-TAVI readmission rates are variable but high. All-cause early readmission rates range from 3.5% to 22.4%, and all-cause late readmission rates range from 14.9% to 54.3%. Despite the variability in rehospitalization rates, the causes of hospital readmission are consistent. Noncardiac causes are more common than cardiac causes for readmission. Cardiac causes were responsible for 31.8% to 44.6% of early readmissions, and 32% to 53.7% of late readmissions. Noncardiac causes were responsible for 55.4% to 68.2% of early readmissions, and 46.3% to 68% of late readmissions. The most common noncardiac causes are respiratory and bleeding events, and infection.^5^^,^^8^^,^^13^^,^^14^ The most common cardiac causes for readmission are heart failure and arrhythmia.^5^^,^^8^^,^^13^, ^14^, ^15^

**Table 2:** Early and late readmission rates post-transcatheter aortic valve implantation

Study	Early readmission rates			Late readmission rates		
	All-cause	Cardiac cause	Noncardiac cause	All-cause	Cardiac cause	Noncardiac cause
Arai et al. 2018^13^	42/1215 (3.5)	18/42 (42.9)	24/42 (57.1)	181/1215 (14.9)	59/181 (32.6)	122/181 (67.4)
Auffret et al. 2020^16^	—	—	—	301/750 (40.1)	138/301 (45.8)∗	—
Czarnecki et al. 2019^28^	157/937 (16.8)	—	—	462/937 (49.3)	—	—
Czarnecki et al. 2020^15^	396/2547 (15.5)	—	—	1170/2547 (45.9)	—	—
Dodson et al. 2017^23^	(17.9)†	—	—	—	—	—
Durand et al. 2017^17^	—	—	—	285/546 (52.2)	—	—
Elbaz-Greener et al. 2019^29^	327/2129 (15.4)	104/327 (31.8)	223/327 (68.2)	924/2129 (44.2)	296/924 (32)	628/924 (68)
Forcillo et al. 2017^18^	74/714 (10.4)	33/74 (44.6)	41/74 (55.4)	134/714 (18.8)	72/134 (53.7)	62/134 (46.3)
Franzone et al. 2017^8^	—	—	—	221/868 (25.4)	142/308 (46.1)	166/308 (53.8)
Guedeney et al. 2019^19^	—	—	—	—	—	—
Kolte et al. 2017^14^	2188/12,221 (17.9)	836/2188 (38.2)	1352/2188 (61.8)	—	—	—
Nombela-Franco et al. 2015^5^	115/720 (16)	49/115 (42.6)	66/115 (57.4)	391/720 (54.3)	159/391 (40.7)	232/391 (59.3)
Panaich et al. 2016^4^	1215/5433 (22.4)	439/1215 (36.2)∗	776/1215 (63.8)∗	—	—	—
Sanchez et al. 2020^30^	950/10,345 (9.2)	—	—	—	—	—
Tripathi et al. 2020^31^	—	—	—	16,343/73,784 (22.2)	5294/16,343 (36.2)	10,419/16,343 (63.8)
Yerasi et al. 2021^32^	269/3104 (8.6)	—	—	—	—	—
Doshi et al. 2019^24^	4532/54,117 (17.2)	—	—	—	—	—

Values are n/N (%), unless otherwise indicated.

∗ Calculated from study data.

† Study reported a median 30-day readmission rate.

### Mortality rates

Eight studies (35,552 patients) reported the all-cause mortality rate, which ranged from 1.1% to 44.4%, with the mean follow-up duration ranging from 1 to 32 months (Table 3). Four studies (14,794 patients) reported mortality rate in readmitted patients, and 2 studies (1859 patients) reported mortality rate in non-readmitted patients. The mortality rate in readmitted patients was higher (30.2%), compared with that in patients without hospital readmission (19.2%). Multiple studies reported a statistically significant increase in all-cause mortality in readmitted patients, compared with that in patients who were not readmitted.^5^^,^^16^, ^17^, ^18^, ^19^ Mortality risk also correlated with number of hospital readmissions and time of readmission. Patients with multiple hospital readmissions or a late readmission were at higher risk for mortality compared to patients with a single or early readmission.^16^^,^^17^

**Table 3:** All-cause mortality rate, and mortality rate in readmitted and non-readmitted patients post-transcatheter aortic valve implantation

Study	Mean follow-up period, mo	All-cause mortality rate	Mortality rate in readmitted patients	Mortality rate in non-readmitted patients
Auffret et al. 2020^16^	32	333/750 (44.4)	—	—
Czarnecki et al. 2019^28^	12	126/937 (13.4)	—	—
Czarnecki et al. 2020^15^	12	268/2547 (10.5)	—	—
Dodson et al. 2017^23^	1	201/18,568 (1.1)	—	—
Durand et al. 2017^17^	27.2	172/546 (31.5)∗	—	—
Forcillo et al. 2017^18^	—	—	36/208 (17)	—
Guedeney et al. 2019^19^	12	145/1139 (12.9)	22/99 (22.2)	(12)
Kolte et al. 2017^14^	—	—	109/2188 (5)	—
Nombela-Franco et al. 2015^5^	24	150/720 (20.8)∗	35/115 (30.2)∗	106/605 (19.2)∗
Sanchez et al. 2020^30^	1	12/616 (1.9)	—	—

Values are n/N (%), unless otherwise indicated.

∗ Calculated from study data.

### Predictors for early hospital readmission

**Figure 2:**
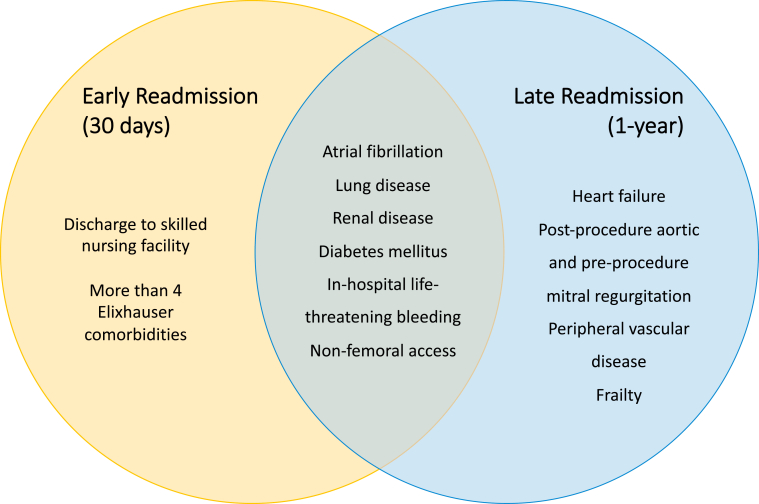
The most consistently identified predictors for early and late readmission post-transcatheter aortic valve implantation. Elixhauser indicates a comorbidity among those used to determine the Elixhauser Comorbidity Index.

Of the 49 included studies, 18 assessed risk factors for early hospital readmission post-TAVI. In Figure 2, we summarize the most frequently identified predictors for early readmission, and a list of all identified predictors can be found in Supplemental Table S4. A total of 37 unique predictors were identified among the 18 studies. Across the 37 predictors, the most-reported risk factor was atrial fibrillation/flutter (5 studies; 55,085 patients). The next most common predictors (4 studies each) were discharge to a skilled nursing facility (68,282 patients), chronic lung disease (39,595 patients), and non-femoral access (38,620 patients). The next most reported risk factors were as follows: diabetes mellitus (3 studies; 34,615 patients); in-hospital life-threatening bleeding, vascular complication, or transfusion (3 studies; 66,543 patients); and more than 4 Elixhauser comorbidities (2 studies; 12,562 patients). Taken together, renal pathologies (acute kidney injury, chronic kidney disease, and renal failure) were identified by 10 studies (96,625 patients) as risk factors for readmission post-TAVI. In the demographic factors category, the most identified risk factor for readmission was increasing age (3 studies; 74,814 patients). For laboratory markers, 2 studies (1434 patients) found an association between hemoglobin level and rehospitalization risk. A low hemoglobin level at discharge increased risk for readmission, whereas a high preoperative hemoglobin level was protective against readmission.

### Predictors for late hospital readmission

Of the 49 included studies, 34 identified risk factors for late hospital readmission post-TAVI. Figure 2 depicts the most frequently identified predictors for late readmission, and a list of all identified predictors can be found in Supplemental Table S5. Across the 34 studies, a total of 59 unique predictors were reported. The predictor most frequently identified by the included studies was atrial fibrillation (11 studies; 157,601 patients)—both pre-existing and new-onset atrial fibrillation. Other cardiac risk factors included heart failure (5 studies; 79,563 patients) and post-TAVI aortic and pre-TAVI mitral regurgitation (4 studies; 37,408 patients). Among the medical comorbidities category, the most common predictors were those related to lung pathology (including chronic obstructive pulmonary disease, interstitial lung disease, pulmonary hypertension and chronic lung disease [7 studies; 8821 patients]), diabetes mellitus (3 studies; 75,673 patients), renal pathology (including chronic kidney injury, acute kidney injury, and stage 3 kidney injury [5 studies; 78,631 patients]), or peripheral vascular disease (3 studies; 3786 patients). Frailty (3 studies; 26,550 patients) and increased Charlson score (2 studies; 4676 patients) were the most common clinical risk factors, and in-hospital life-threatening bleeding, vascular complications, or transfusions was the most common procedural complication risk factor identified (4 studies; 76,135 patients). Increased age, male sex, anemia, nonfemoral access, and discharge to a skilled nursing facility remained predictors for readmission.

## Discussion

The purpose of this systematic review was to identify predictors of both early and late readmission in patients post-TAVI. Our main findings are as follows: (i) post-TAVI readmission is common and has both cardiac and noncardiac causes; (ii) a possible association exists between post-TAVI readmission and subsequent mortality, requiring further investigation; and (iii) several comorbidities and procedural variables predict post-TAVI readmission with significant overlap between predictors for early and late readmission. These findings highlight the importance of identifying patients at elevated risk for post-TAVI readmission who may benefit from additional management or follow-up post-procedure; however, the current lack of tools available to identify patients most at risk for readmission may make this challenging. The ability to stratify patients according to readmission risk is necessary because initiatives aimed at lowering rehospitalization rates may be more successful when targeted toward those patients who are most at risk.^20^

Similar work in this area has been done by Goldsweig et al. (2020)^21^ and Li et al. (2021)^22^ Goldsweig et al.^21^ conducted a rapid review to identify predictors for post-TAVI readmission. They reported on 10 studies and 24 unique predictors. In their systematic review, Li et al.^22^ also reported on 10 studies and found a total of 15 unique predictors. Both of these studies identify the following predictors as being the most common: non-transfemoral valve delivery, kidney disease, lung disease, major bleeding, atrial fibrillation, and left ventricular systolic dysfunction. Our current work builds on the findings of both these previous reviews. We performed a more comprehensive systematic search encompassing studies published to date. In comparison, Li et al.^22^ searched only the literature up to 2018 and found 13 studies. As a result, compared to both these previous reviews, we were able to identify substantially more relevant articles at 49. In addition, our search identified several novel predictors commonly reported in the literature— heart failure, post-procedure aortic and pre-procedure mitral regurgitation, diabetes mellitus, peripheral vascular disease, frailty, and discharge to a skilled nursing facility. We also report on factors found to be protective against readmission, such as a high preoperative hemoglobin level and the absence of anemia prior to TAVI. Lastly, we present mortality data demonstrating an increased risk for mortality in TAVI patients with a post-procedure readmission, compared to the risk in those without one. This association may simply reflect the fact that the comorbidities that drive mortality and readmission are likely the same; moreover, this finding is limited, as only a small number of all included studies present mortality data.

The evidence is conflicting for several predictors, such as female sex, heart failure, valve type, pacemaker implantation, left bundle branch block, age, coronary artery disease, and post-procedure aortic regurgitation. Dodson et al. (2017)^23^ found female sex to be protective against post-procedure readmission, whereas Doshi et al. (2019)^24^ found female sex to increase the likelihood of post-procedure readmission. Similarly, multiple studies have shown that nonagenarians have a risk level for post-procedure readmission similar to that of a younger population. Moreover, we found one study that revealed an association of balloon-expanding valves with a decrease in readmission risk, compared with the risk with self-expanding valves, but this association was not found in the Comparison of Balloon-Expandable vs Self-expandable Valves in Patients Undergoing Transcatheter Aortic Valve Replacement (CHOICE)^25^ and Repositionable Percutaneous Replacement of Stenotic Aortic Valve Through Implantation of Lotus Valve System—Randomized Clinical Evaluation (REPRISE III)^26^ RCTs. In addition, we found evidence for increased readmission risk in patients requiring pacemaker implantation, but once more, this association was not seen in the REPRISE III RCT.^26^ Given the large number of studies, we believe the value of our work is in summarizing the breadth of the literature and identifying those risk factors for which the findings show broad consistency.

Our findings have significant implications with respect to post-TAVI readmission rates. By identifying common predictors for early and late post-procedure readmission, our work can inform risk stratification. This benefit will aid both primary care physicians and interdisciplinary TAVI teams, allowing them to follow high-risk patients more closely, which may reduce rehospitalization risk. Moreover, future projects may focus on developing and evaluating interventions to reduce readmission rates among the high-risk cohort. Decreased post-TAVI readmission rates may benefit both patients—through reduced risk for mortality and morbidity—and the healthcare system—through reduced costs from rehospitalizations.^8^^,^^9^ In the context of heart failure, post-discharge remote monitoring has proven effective at identifying early health deterioration, allowing for prompt intervention to prevent rehospitalization.^27^ As part of our future research, we plan to apply this strategy to TAVI patients at high risk for readmission at our centre.

### Limitations

Our study findings should be evaluated in the context of several limitations. First, we did not explore studies published prior to 2015. Although this strategy could have resulted in oversight of relevant studies, we believe this to be unlikely given that our initial literature search found that the earliest relevant paper was published in 2015. Additionally, by restricting our search to this timeframe, our results are more in line with current TAVI practices and patient populations, enhancing their generalizability in the modern era. And second, our systematic review study design is limited in that it does not provide a summary estimate for our primary or secondary outcomes, as would have been possible with a meta-analysis design. We deemed a qualitative review to be more appropriate given the substantial heterogeneity among studies with regard to populations and reporting of outcome data.

## Conclusion

This review demonstrates that 30-day and 1-year readmission rates post-TAVI are high, and that increased mortality is associated with readmission. In addition, it identifies the most common risk factors for both 30-day and 1-year hospital readmission. The results of this study can be used to identify patients at high-risk for hospital readmission post-TAVI. Tailored strategies can be developed accordingly to reduce readmission rates in high-risk cohorts.
