# Supplementary material for: Risk Factors for Hospital Readmission Post-Transcatheter Aortic Valve Implantation in the Contemporary Era: A Systematic Review
Source: CJC Open. 2022 Jun 6;4(9):792–801. doi: 10.1016/j.cjco.2022.05.007 (PMC9486870; doi:10.1016/j.cjco.2022.05.007)
Supplement: Supplemental Tables S1-S5 [file mmc1.pdf]

SUPPLEMENTARY MATERIAL

Supplemental Table S1. Full search strategies

Database: Ovid MEDLINE(R) and Epub Ahead of Print, In-Process, In-Data-Review & Other Non-Indexed Citations, Daily and Versions(R) <1946 to March 19, 2021> Search Strategy:

----

1 exp Transcatheter Aortic Valve Replacement/ (6663)

2 ((transcatheter or transapical or transfemoral or transcutaneous or transvascular or percutaneous) adj5 aortic adj5 valve* adj5 (implant* or replace*)).mp. (12861)

3 (TAVI or TAVR or PAVI or PAVR).mp. (8599)

4 or/1-3 (13493)

5 exp Patient Readmission/ (18764)

6 (readmission* or re-admission*).mp. (38940)

7 (readmit* or re-admit*).mp. (8499)

8 (rehospitali* or re-hospitali*).mp. (8272)

9 or/5-8 (48021)

10 4 and 9 (436)

11 limit 10 to english language (425)

12 limit 11 to yr="2015 -Current" (395)

Database: Embase Classic+Embase <1947 to 2021 Week 11> Search Strategy:

----

1 exp transcatheter aortic valve implantation/ (23875)

2 ((transcatheter or transapical or transfemoral or transcutaneous or transvascular or percutaneous) adj5 aortic adj5 valve* adj5 (implant* or replace*)).tw,kw. (20999)

3 (TAVI or TAVR or PAVI or PAVR).tw,kw. (18667)

4 or/1-3 (27668)

5 exp hospital readmission/ (71972)

6 (readmission* or re-admission*).tw,kw. (61492)

7 (readmit* or re-admit*).tw,kw. (18476)

8 (rehospitali* or re-hospitali*).tw,kw. (15255)

9 or/5-8 (98102)

10 4 and 9 (1284)

11 limit 10 to english language (1272)

12 limit 11 to yr="2015 -Current" (1113)

Database: EBM Reviews - Cochrane Central Register of Controlled Trials <February 2021>

Search Strategy:

----

1 exp Heart Valve Prosthesis Implantation/ (680)

2 ((transcatheter or transapical or transfemoral or transcutaneous or transvascular or percutaneous) adj5 aortic adj5 valve* adj5 (implant* or replace*)).mp. (1109)

3 (TAVI or TAVR or PAVI or PAVR).mp. (1019)

4 or/1-3 (1776)

5 exp Patient Readmission/ (1072)

6 (readmission* or re-admission*).mp. (7943)

7 (readmit* or re-admit*).mp. (811)

8 (rehospitali* or re-hospitali*).mp. (2607)

9 or/5-8 (9701)

10 4 and 9 (83)

11 limit 10 to english language (73)

12 limit 11 to yr="2015 -Current" (58)

Supplemental Table S2. Data elements extracted from included studies

Outcome variables	Report variables	Study variables	Patient variables	Intervention variables
Predictors for early readmission	Title	Country	Average age	Transfemoral access (%)
Predictors for late readmission	Year	Design	Female patients (%)	Balloon-expandable valve (%)
All-cause early readmission rate	First author	Period	STS Score	Self-expandable valve (%)
All-cause late readmission rate	Journal	TAVI registry	EuroSCORE	Another valve (%)
Cardiac cause early readmission rate		Follow-up period	EuroSCORE II	
Non-cardiac cause early readmission rate		Inclusion and exclusion criteria		
Non-cardiac cause late readmission rate				
All-cause mortality rate				
Mortality rate in readmitted patients				

The participant and intervention data were collected for each study cohort (overall cohort, cohort of patients with readmissions, cohort of patients without readmissions).

Supplemental Table S3. Quality assessment of included studies

Study	Selection				Comparability		Outcome			Total Score
	Representative of the exposed cohort	Selection of the external control	Ascertainment of exposure	Outcome of interest not present at start of study	Main factor	Additional factor	Assessment of outcomes	Sufficient follow-up time	Adequacy of follow-up	
Arai et al. 2018	1	1	1	1	1	1	1	1	1	9
Auffret et al. 2020	1	1	1	1	1	1	1	1	1	9
Czarnecki et al. 2019	1	1	1	1	1	1	1	1	1	9
Czarnecki et al. 2020	1	1	1	1	1	1	1	1	1	9
Dodson et al. 2017	1	1	1	1	1	1	1	1	1	9
Durand et al. 2017	1	1	1	1	1	1	1	1	1	9
Elbaz-Greener et al. 2019	1	1	1	1	1	1	1	1	1	9
Forcillo et al. 2017	1	1	1	1	1	1	0	1	1	8
Franzone et al. 2017	1	1	1	1	1	1	0	1	1	8
Guedeney et al. 2019	1	1	1	1	1	1	1	1	1	9
Kolte et al. 2017	1	1	1	1	1	1	1	1	1	9
Nombela-Franco et al. 2015	1	1	1	1	1	1	0	1	1	8
Panaich et al. 2016	1	1	1	1	1	1	1	1	1	9
Sanchez et al. 2020	1	1	1	1	1	1	1	1	1	9
Tripathi et al. 2020	1	1	1	1	1	1	1	1	1	9
Yerasi et al. 2021	1	1	1	1	1	1	1	1	1	9

Supplemental Table S4. Predictors for early hospital readmission post-TAVI

Predictor	Measure of Effect	95% Confidence Interval	P-value	Study	Total number of predictors in category
**Demographic factors**					
Female sex	OR 0.91	0.84-0.99	0.03	Dodson et al. 2017	2
	OR 1.09	1.01-1.19	< 0.001	Doshi et al. 2019	
Age	OR 1.00	1.00-1.01	0.04	Dodson et al. 2017	
	HR 1.02	1.00-1.04	0.02	Elbaz-Greener et al. 2019	
**Clinical characteristic**					
New York Heart Association class III/IV	OR 1.12	1.00-1.25	0.04	Dodson et al. 2017	9
	OR 1.33	1.01-1.76	0.043	Sanchez et al. 2020	
Charlson score	HR 1.12	1.02-1.22	0.02	Elbaz-Greener et al. 2019	
Length of stay > 5 days	HR 1.47	1.24-1.73	< 0.001	Kolte et al. 2017	
	OR 1.65	1.48-1.83	-	Arora et al. 2020	
Length of stay > 10 days	OR 3.06	2.22-4.22	< 0.01	Panaich et al. 2016	
> 4 Elixhauser comorbidities	HR 1.22	1.03-1.46	0.026	Kolte et al. 2017	
	OR 1.21	1.02-1.44	-	Feldman et al. 2021	
Discharge to skilled nursing facility	OR 1.79	1.62-1.97	< 0.001	Yerasi et al. 2021	
	HR 1.16	1.01-1.34	0.038	Kolte et al. 2017	
	OR 1.14	1.01-1.28	-	Arora et al. 2020	
	OR 1.28	1.14-1.43	< 0.01	Panaich et al. 2016	
Single or dual anti-platelet +	OR 1.62	1.10-2.39	0.014	Nombela-Franco et al. 2015	

anticoagulation therapy					
Home oxygen use	OR 1.38	1.04-1.82	0.025	Sanchez et al. 2020	
Malnourishment	OR 1.34	-	<0.001	Emami et al. 2020	
**Cardiac comorbidity or intervention**					
Atrial fibrillation/flutter	OR 1.26	1.17-1.36	< 0.001	Dodson et al. 2017	6
	HR 1.23	1.09-1.40	0.001	Kolte et al. 2017	
	OR 1.66	1.37-2.02	< 0.001	Sanchez et al. 2020	
	OR 1.42	1.31-1.54	< 0.001	Yerasi et al. 2021	
	OR 1.39	1.28-1.51	< 0.001	Doshi et al. 2020	
Prior cardiac surgery	OR 0.85	0.77-0.94	0.0015	Dodson et al. 2017	
Permanent pacemaker placement	OR 1.28	0.47-0.97	0.04	Panaich et al. 2016	
Post-procedural aortic regurgitation (moderate)	OR 1.64	1.06-2.54	0.026	Sanchez et al. 2020	
Conduction disease	OR 1.18	1.08-1.29	< 0.001	Yerasi et al. 2021	
Heart failure	OR 1.18	1.07-1.30	< 0.001	Yerasi et al. 2021	
**Medical comorbidity**					
Acute kidney injury	HR 2.27	1.04-4.94	0.03	Arai et al. 2018	10
	HR 1.23	1.05-1.44	0.011	Kolte et al. 2017	
	OR 1.59	1.39-1.81	-	Arora et al. 2020	
	-	-	< 0.0001	Thourani et al. 2016	

Chronic lung disease	OR 1.22	1.12-1-33	< 0.001	Dodson et al. 2017
	OR 1.32	1.18-1.47	< 0.01	Panaich et al. 2016
	HR 1.16	1.01-1.34	0.034	Kolte et al. 2017
	OR 1.24	1.14-1.35	< 0.001	Yerasi et al. 2021
Pulmonary hypertension > 60 mmHg	-	-	< 0.03	Testa et al. 2016
Diabetes mellitus	OR 1.13	1.04-1-1.23	0.0034	Dodson et al. 2017
	OR 1.18	1.06-1.32	0.004	Panaich et al. 2016
	OR 1.24	1.02-1.51	0.03	Sanchez et al. 2020
Stroke/transient ischemic attack	OR 1.47	1.13-1.89	0.0034	Dodson et al. 2017
Anemia	HR 0.78	0.62-0.99	-	Forcillo et al. 2017
	OR 1.30	1.10-1.54	0.002	Yerasi et al. 2021
Chronic kidney disease	HR 1.20	1.04-1.39	0.014	Kolte et al. 2017
	OR 1.42	1.31-1.54	< 0.001	Yerasi et al. 2021
	OR 2.10	1.02-4.32	0.04	Gracia et al. 2020
Chronic dialysis	OR 1.97	1.64-2.40	-	Ando et al. 2020
Renal failure	OR 1.43	1.24-1.65	< 0.01	Panaich et al. 2016
Neoplastic disorders	OR 1.28	1.07-1.54	0.007	Yerasi et al. 2021
**Laboratory marker**				
Glomerular filtration rate (< 30 vs ≥ 30)	OR 1.33	1.14-1.56	< 0.001	Dodson et al. 2017
Glomerular filtration rate (on dialysis vs ≥ 30)	OR 1.61	1.34-1.93	< 0.001	Dodson et al. 2017

Preoperative hemoglobin, g/dl	HR 0.74	0.59-0.93	-	Forcillo et al. 2017	
Hemoglobin at hospital discharge, g/dl (for each decrease of 1 g/dl)	OR 1.19	1.03-1.39	0.019	Nombela-Franco et al. 2015	
Post-procedural creatinine > 1.6 mg/dL	OR 1.53	1.21-1.92	0.001	Sanchez et al. 2020	
**Procedural characteristic**					
Nonfemoral access	OR 1.43	1.31-1.57	< 0.001	Dodson et al. 2017	4
	HR 2.33	1.82-2.99	< 0.0001	Elbaz-Greener et al. 2019	
	HR 1.21	1.05-1.39	0.008	Kolte et al. 2017	
	OR 1.23	1.10-1.38	< 0.01	Panaich et al. 2016	
Endovascular vs. transapical	OR 0.83	0.70-0.98	0.036	Lemor et al. 2019	
Valve size – 23 mm	HR 1.64	1.02-2.63	-	Forcillo et al. 2017	
Left ventricular ejection fraction at hospital discharge (for each decrease of 5%)	OR 1.08	1.00-1.17	0.042	Nombela-Franco et al. 2015	
**Procedural complication**					

In-hospital life-threatening bleeding, vascular complication, or transfusion	OR 1.30	1.19-1.42	< 0.001	Dodson et al. 2017	1
	OR 1.34	1.18-1.53	< 0.001	Dodson et al. 2017	
	OR 2.41	1.57-3.70	< 0.001	Nombela-Franco et al. 2015	
	OR 1.49	1.25-1.70	-	Arora et al. 2020	
	OR 1.23	1.07-1.40	-	Arora et al. 2020	

Supplemental Table S5. Predictors for late hospital readmission post-TAVI

Predictor	Measure of Effect	95% Confidence Interval	P-value	Study	Total number of predictors in category
**Demographic factor**					
Female sex	HR 0.72	0.53-0.98	0.03	Czarnecki et al. 2019	3
	OR 1.09	1.05-1.14	< 0.001	Pajjuru et al. 2022	
Male sex	SHR 1.33	1.02-1.73	0.035	Franzone et al. 2017	
Age ≥ 90	OR 1.22	1.12-1.32	0.001	Tripathi et al. 2020	
**Clinical characteristic**					
Frailty	HR 1.22	1.03-1.81	0.02	Arai et al. 2018	13
	RR 1.04	1.04-1.05	< 0.001	Czarnecki et al. 2020	
	HR 1.24	1.05-1.48	0.01	Elbaz-Greener et al. 2019	
	HR 1.68	1.13-2.50	0.010	Saji et al. 2018	
	-	-	< 0.01	Malik et al. 2020	

New York Heart Association class IV	HR 1.43	1.07-1.91	0.02	Czarnecki et al. 2019
Post-TAVI rehabilitation	HR 1.34	1.11-1.62	0.002	Czarnecki et al. 2019
Post-TAVI cardiologist follow-up	HR 1.41	1.14-1.75	0.002	Czarnecki et al. 2019
Charlson score	RR 1.12	1.09-1.16	< 0.001	Czarnecki et al. 2020
	HR 1.10	1.04-1.16	0.001	Elbaz-Greener et al. 2019
Low aortic mean gradient before TAVI	HR 0.88	0.79-0.99	0.03	Durand et al. 2017
Left atrium diameter post-TAVI	HR 1.47	1.08-2.01	0.02	Durand et al. 2017
Length of hospital stay	HR 1.14	1.01-1.29	-	Forcillo et al. 2017
Length of hospital stay > 2 days vs ≤ 2 days	OR 1.40	1.32-1.49	< 0.001	Tripathi et al. 2020
Prior stroke	OR 1.15	1.07-1.24	< 0.001	Tripathi et al. 2020
Prior pacemaker placement	OR 1.09	1.01-1.17	0.022	Tripathi et al. 2020
Discharge to skilled nursing facility	OR 1.58	1.47-1.69	< 0.001	Tripathi et al. 2020
Patient given prescription for RAS inhibitor	OR 0.86	0.79-0.95	-	Inohara et al. 2018
**Cardiac comorbidity or intervention**				

Atrial fibrillation	HR 1.70	1.26-2.30	< 0.01	Arai et al. 2018
	HR 1.34	1.08-1.66	0.01	Czarnecki et al. 2019
	RR 1.25	1.17-1.35	< 0.001	Czarnecki et al. 2020
	HR 1.47	1.27-1.70	< 0.001	Elbaz-Greener et al. 2019
	adjHR 1.62	1.09-2.40	-	Guedeney et al. 2019
	OR 1.32	1.06-1.63	0.012	Nombela-Franco et al. 2015
	OR 1.39	1.32-1.46	< 0.001	Tripathi et al. 2020
	HR 4.04	2.23-7.32	-	Hioki et al. 2017
	HR 1.80	-	0.015	Shahim et al. 2021
	-	-	0.04	Zweiker et al. 2017
	HR 1.77	1.67-1.86	< 0.0001	Mentias et al. 2019
Postprocedural aortic regurgitation (mild)	HR 1.62	1.22-2.14	< 0.01	Arai et al. 2018
Previous acute heart failure	SHR 1.69	1.05-2.73	0.03	Auffret et al. 2020

Aortic regurgitation grade III/IV at discharge	SHR 7.19	3.07-16.38	<0.001	Auffret et al. 2020
Left ventricular dysfunction	HR 0.73	0.54-0.98	0.04	Czarnecki et al. 2019
Left bundle branch block	OR 1.57	1.28-2.07	0.002	Jorgensen et al. 2019
	OR 1.66	1.11-2.50	0.02	Nazif et al. 2019
Pre-TAVI Mitral regurgitation (mild)	HR 1.23	1.10-1.38	< 0.001	Czarnecki et al. 2019
Pre-TAVI Mitral regurgitation (moderate/severe)	HR 1.26	1.03-1.54	0.03	Czarnecki et al. 2019
	HR 1.95	1.20-3.15	0.007	Miura et al. 2020
	HR 1.19	1.10-1.30	< 0.001	McCarthy et al. 2017
Post-TAVI lack of improvement in mitral regurgitation	HR 1.50	1.01-2.22	0.044	Freitas-Ferraz et al. 2020
Chronic heart failure	RR 1.45	1.31-1.60	< 0.001	Czarnecki et al. 2020
	HR 1.30	1.01-1.55	< 0.003	Elbaz-Greener et al. 2019
	HR 2.43	1.19-4.95	0.015	Johansson et al. 2016
	OR 1.17	1.10-1.24	0.001	Tripathi et al. 2020

Coronary artery disease	RR 1.17	1.07-1.29	< 0.001	Czarnecki et al. 2020
Prior percutaneous coronary intervention	RR 1.11	1.02-1.19	0.01	Czarnecki et al. 2020
Previous myocardial infarction	SHR 1.88	1.22-2.90	0.004	Franzone et al. 2017
Post-TAVI permanent pacemaker implementation	-	-	0.05	Nazif et al. 2015
	HR 1.42	1.06-1.89	0.019	
	HR 1.28	1.15-1.43	< 0.001	Aljabbary et al. 2018
**Medical comorbidity**				
Anemia	HR 2.21	1.61-3.25	< 0.01	Arai et al. 2018
	OR 1.13	1.06-1.22	< 0.001	Tripathi et al. 2020
Chronic lung disease	SHR 1.88	1.20-2.94	0.006	Auffret et al. 2020
Diabetes mellitus orally treated	SHR 2.14	1.27-3.63	0.004	Auffret et al. 2020
Diabetes mellitus Insulin-treated	SHR 2.35	1.17-4.73	0.017	Auffret et al. 2020
Diabetes mellitus	adjHR 1.67	1.11-2.50	-	Guedeney et al. 2019
	OR 1.15	1.09-1.21	< 0.001	Tripathi et al. 2020
Pulmonary hypertension at discharge (moderate/severe)	SHR 2.22	1.26-3.92	0.006	Auffret et al. 2020

	SHR 4.68	1.93-11.35	0.001	Auffret et al. 2020
	HR 1.04	1.00-1.07	< 0.0001	Durand et al. 2017
Peripheral vascular disease	HR 1.18	1.02-1.37	0.02	Czarnecki et al. 2019
	HR 1.44	1.13-1.83	< 0.001	Elbaz-Greener et al. 2019
	OR 1.29	1.04-1.61	0.023	Nombela-Franco et al. 2015
Dialysis	HR 1.67	1.06-2.65	0.03	Czarnecki et al. 2019
Peptic ulcer disease	HR 1.52	1.09-2.12	0.01	Czarnecki et al. 2019
In-hospital infection	HR 2.00	1.27-3.14	-	Tirado-Conte et al. 2016
Chronic obstructive pulmonary disease	RR 1.16	1.08-1.25	< 0.001	Czarnecki et al. 2020
	HR 1.22	1.07-1.40	0.004	Elbaz-Greener et al. 2019
	adjHR 1.81	1.17-2.81	-	Guedeney et al. 2019

	HR 1.49	1.21-1.84	< 0.001	Nombela-Franco et al. 2015
Interstitial lung disease	RR 1.28	1.05-1.56	0.01	Czarnecki et al. 2020
Pulmonary hypertension > 60 mmhg	-	-	< 0.03	Testa et al. 2016
Stage 3 kidney injury	SHR 2.04	1.12-3.71	0.021	Franzone et al. 2017
	-	-	< 0.0001	Thourani et al. 2016
Chronic kidney disease	adjHR 1.72	1.13-2.62	-	Guedeney et al. 2019
	OR 1.33	1.26-1.40	< 0.001	Tripathi et al. 2020
Liver disease	OR 1.24	1.01-1.53	0.038	Tripathi et al. 2020
Acute kidney injury	OR 1.20	1.11-1.29	< 0.001	Tripathi et al. 2020
End stage liver disease and end stage renal disease	-	-	0.02	Caughron et al. 2021
**Laboratory marker**				
Albumin < 3.5 mg/dl	HR 1.37	1.01-1.86	0.04	Arai et al. 2018
Hemoglobin g/L	HR 0.99	0.99-1.00	< 0.001	Czarnecki et al. 2019

Glomerular filtration rate at hospital discharge ml/min	OR 1.05	1.01-1.09	0.013	Nombela-Franco et al. 2015	
Increase of 100 pg/mL in BNP	HR 1.08	1.03-1.14	-	O'leary et al. 2020	
Post-TAVI intravascular hemolysis	OR 4.50	1.30-15.6	0.02	Ko et al. 2018	
**Procedural characteristic**					
Valve-in-valve TAVI	HR 0.65	0.48-0.87	0.004	Czarnecki et al. 2019	
	RR 0.82	0.70-0.96	0.01	Czarnecki et al. 2020	
Nonfemoral access	HR 1.40	1.08-1.81	0.01	Czarnecki et al. 2019	4
	HR 1.46	1.23-1.74	< 0.001	Elbaz-Greener et al. 2019	
	OR 1.19	1.03-1.38	0.019	Tripathi et al. 2020	
Left ventricular ejection fraction post-TAVI $\leq$ 35%	adjHR 2.12	1.20-3.75	-	Guedeney et al. 2019	
Balloon-expandable valve	OR 0.84	0.78-0.90	< 0.0001	Deharo et al. 2020	
**Procedural complication**					
In-hospital life-threatening bleeding, vascular complication, or transfusion	SHR 2.18	1.24-3.85	0.007	Franzone et al. 2017	2
	OR 1.16	1.05-1.27	0.002	Tripathi et al. 2020	

	HR 1.33	1.18-1.50	< 0.001	Czarnecki et al. 2019
	HR 2.27	1.13-5.56	0.009	Durand et al. 2017
Patient-prosthesis mismatch	HR 1.12	1.02-1.24	0.017	Hermann et al. 2018
